# A Modified Pre-plating Method for High-Yield and High-Purity Muscle Stem Cell Isolation From Human/Mouse Skeletal Muscle Tissues

**DOI:** 10.3389/fcell.2020.00793

**Published:** 2020-08-13

**Authors:** Kiyoshi Yoshioka, Yasuo Kitajima, Narihiro Okazaki, Ko Chiba, Akihiko Yonekura, Yusuke Ono

**Affiliations:** ^1^Department of Muscle Development and Regeneration, Institute of Molecular Embryology and Genetics, Kumamoto University, Kumamoto, Japan; ^2^Department of Orthopaedic Surgery, Nagasaki University Graduate School of Biomedical Sciences, Nagasaki, Japan

**Keywords:** skeletal muscle, satellite cell, muscle stem cell, isolation, pre-plating

## Abstract

Primary culture of skeletal muscle stem cells (MuSCs) is indispensable to study the dynamics of muscle regeneration and homeostasis. Here we describe the modified pre-plating method for isolating MuSCs in culture with greatly improved purity, yield, and procedure time. The protocol is based on the distinct adhesion characteristics of MuSCs. We reduced the procedure time to 2.5 days to obtain highly purified MuSCs through a newly employed re-plating step, which repeats incubation and cell-suspension. The re-plating step efficiently traps remaining fibroblastic cells, but not MuSCs, on a collagen-coated dish. Additionally, we confirmed that MuSCs can be isolated from small amounts of human/mouse muscle tissues, enabling us to perform experiments with amount-limited specimens. Thus, our method can be performed with basic laboratory equipment suitable for most facilities and without sophisticated MuSC handling techniques.

## Introduction

Adult skeletal muscle stem cells (MuSCs), also known as muscle satellite cells, are the resident tissue stem cells located between the basal lamina and the plasmalemma of myofibers (Mauro, 1961). MuSCs play important roles in muscle growth, hypertrophy repair, and regeneration in adults (Sacco et al., 2008; Relaix and Zammit, 2012; Yin et al., 2013; Cornelison, 2018; Kitajima et al., 2018). Postnatal myogenesis entails quiescent satellite cell activation, myoblast (activated satellite cells) proliferation, and the fusion of myoblasts into multinucleated fibers (Morgan and Partridge, 2003). Myogenesis is a complex process regulated by transcription factors, including myogenic regulatory factors (MRFs) (Parker et al., 2003; Wagers and Conboy, 2005; Braun and Gautel, 2011). MuSCs are essential for skeletal muscle homeostasis and regeneration throughout life (Blau et al., 2015). Thus, understanding the dynamics of MuSCs can provide valuable insights into the mechanisms of muscle degeneration in disease and in aging.

Fluorescence-activated cell sorting (FACS) is a well-established method that has been widely used to isolate MuSCs. The procedure usually involves enzymatic digestion followed by antibody staining for MuSC-specific markers (e.g., α7-integrin, Vcam1, CD34, and CD82) and negative markers (e.g., CD31, CD45, and Sca1) (Montarras et al., 2005; Fukada et al., 2007; Joe et al., 2010; Liu et al., 2013; Alexander et al., 2016; Kitajima et al., 2016; Uezumi et al., 2016). Magnetic cell sorting is an alternate approach to isolate MuSCs. It also requires cell-labeling steps with specific surface markers (Motohashi et al., 2014). Recently, we developed a Pax7-YFP knock-in mouse line that can be directly applied to FACS without immunostaining after enzymatic digestion (Kitajima and Ono, 2018). However, cellular damage during cell sorting is unavoidable given the nature of the method: exposure to high pressure, voltage, and cell-containing droplets striking the liquid surface. These damages are called sorter-induced cellular stress (SICS) (Lopez and Hulspas, 2020). SICS causes cell death, proliferative defect, and potentially influences cellular phenotypes (Andrä et al., 2020). It also has been reported that some fluorescence proteins themselves can be toxic to cells (Liu et al., 1999). Given these limitations, researchers may have experienced unintentional cell-loss during cell-sorting.

Here, we developed a pre-plating technique enabling high yield and high purity MuSC isolation by adding re-plating steps. We have successfully isolated MuSCs from as little as 20 mg of human muscle tissue using this protocol. Our new protocol is ideal to obtain MuSCs from amount-limited samples. The entire process requires 2.5 days, while other pre-plating methods typically require more days to obtain purified MuSCs from whole leg muscles, or needs to fully expand cells in advance to purify (Qu et al., 1998; Jankowski et al., 2001; Park et al., 2006; Gharaibeh et al., 2008; Xu et al., 2018). Finally, the technique does not require expensive equipment or antibodies. Thus, the technique is broadly available and may lead to further progress in skeletal muscle research.

## Materials and Methods

### Muscle Preparation and Enzymatic Dissociation

**FIGURE 1:**
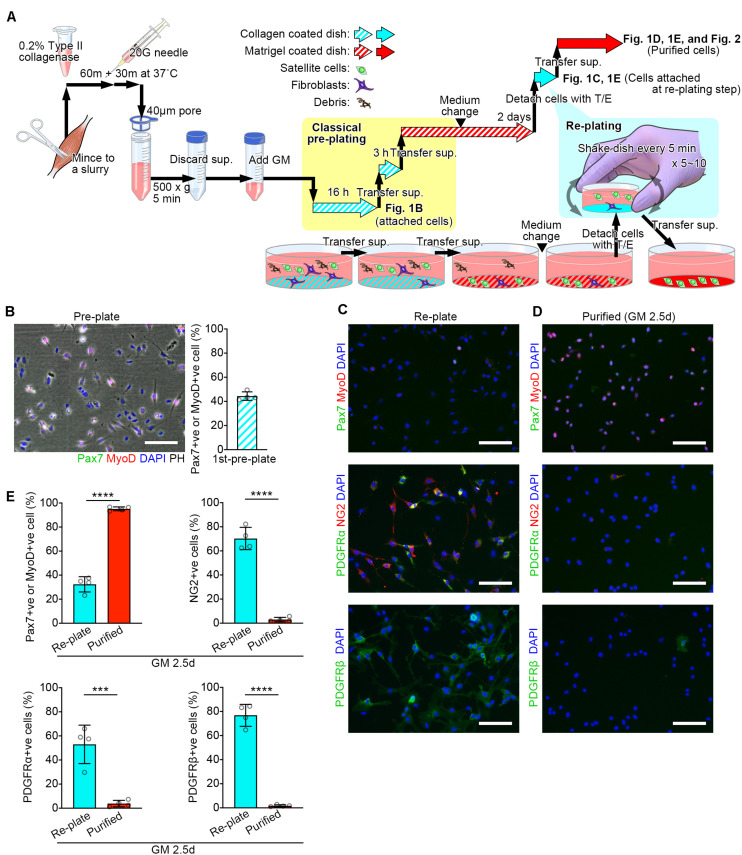
Schematic diagram of the modified pre-plating method. **(A)** Schematic diagram detailed in the methods. **(B–D)** Representative images for cells isolated from mouse lower limb muscles. Scale bars = 100 μm. **(B)** A representative image of attached cells in the collagen coated-dish used for the first pre-plating and its quantification. Cells were stained with Pax7 (green), MyoD (red), and DAPI (blue). Images were merged with phase-contrast images (PH) Error bar = SD, *n* = 4. **(C,D)** Representative images of attached cells in collagen coated-dishes used for the re-plating step **(C)** and purified cells **(D)**. **(E)** Quantification of the cells stained for MuSC markers (Pax7 and MyoD) or non-MuSC markers (NG2, PDGFRα, and PDGFRβ). Error bars = SD, *n* = 4, ^∗∗∗^*p* < 0.001, ^∗∗∗∗^*p* < 0.0001.

Muscle was dissected and collected in phosphate buffered saline (PBS). The muscle was placed in a new petri dish containing PBS. All subsequent manipulations are performed in a culture hood. Any visible fat and nerve deposits were removed with forceps. The muscle was placed on a plastic plate and minced the tissue into a paste with small surgical scissors until no visible muscle deposits remain^∗^. The paste was transferred to a tube contained 0.2% type II collagenase (LS004177, Worthington Biochemical, Freehold, NJ, United States) dissolved in DMEM (Dulbecco’s modified Eagle’s medium, C11995500, Thermo Fisher Scientific, Waltham, MA, United States) containing 1% penicillin streptomycin (PS, 168-23191, Wako, Osaka, Japan). Approximately 3 ml of collagenase solution for 1 g muscle tissue were prepared^∗∗^. The paste was incubated at 37°C for 60 min on a shaker. Pass the minced muscle tissue through a 20G syringe needle several times^∗∗∗^. Incubate at 37°C for 30 min. The homogenate was collected with a 20G syringe needle and dissolved with 40 ml of PBS, then filtered using 40 μm nylon mesh strainer. The flow-through was collected in a 50 ml conical tube and centrifuged at 500 × *g* for 5 min. The supernatant was discarded and the cells were resuspended in growth medium**** (GM, 30% fetal bovine serum (FBS), 1% chick embryo extract (CEE, C3999, USBiological, Swampscott, MA, United States), 10 ng/ml basic fibroblast growth factor (bFGF) and 1% PS in DMEM) (Summarized in Figure 1A).

### Pre-plating, Cell Expansion, and Re-plating

The cell-containing GM was transferred to a type I collagen-coated dish and pre-plated in an incubator overnight (16 h). The next day, the cell-containing GM was pipetted on the dish to suspend un-attached cells. The solution was transferred from the dish into a new type I collagen-coated dish and pre-plated in an incubator for 3 h. The solution was transferred to a plate coated with Matrigel (356234, Corning, Corning, NY, United States) for 24 h. The media was pipetted to suspend debris, then replaced the media with fresh GM and incubated for 24 h. The expanded cells were detached with Trypsin-EDTA (0.25%, 25200072, Thermo Fisher Scientific). The cells were washed with PBS, centrifuged, and resuspended in GM^#^. The cell-containing GM was transferred into a type I collagen-coated dish (4010-010, IWAKI, Tokyo, Japan). The dish was Incubated for 5 min and then gently shaken to suspend un-attached cells^##^. The incubate-and-shake step was repeated 5 times^###^. The solution was transferred to a matrigel-coated plate (Summarized in Figure 1A).

### Tips

*Complete mincing enhances yield of satellite cells. **Because this method enables cell collection from small amounts of tissue, we usually use 300 μl of 0.2% collagenase solution for mouse tibialis anterior (TA) (approximately 50 mg × 2, both sides of the legs). ***Excessive incubation damages the cells. If the slurry smoothly passes 20G syringe needle at this point, further incubation would not be needed. ****Add adequate amount of GM to cover the type I collagen-coated dishes. For two TA mouse muscles, add 4 ml of GM for a 60 mm-dish. For whole leg muscles (approx. 1.5 g), add 7 ml of GM for a 100 mm-dish. ^#^To shak dishes, the amount of GM should be reduced appropriately. ^##^Fibroblasts start to attach to bottom of the collagen-coated dish within 5 min. Do not incubate the dish for more than 5 min. After 5 min, MuSCs start to adhere to the bottom of the dish and longer incubation would lower the yield due to inefficient MuSC transfer at the next re-plating. ^###^The incubate-and-shake step enables further purification of MuSCs. If fibroblasts remain at this step, additional incubate-and-shake steps are recommended.

### Immunostaining for Validation

Immunocytochemistry was performed on satellite cells as previously described (Kitajima et al., 2016). Briefly, samples were fixed with PFA and incubated with primary antibodies at 4°C overnight following blocking/permeabilization with PBS with 0.3% triton X-100 and 5% goat serum for 30 min at room temperature (RT). All immunostaining samples were visualized using appropriate species-specific Alexa Fluor 488 and/or 546 conjugated fluorescent secondary antibodies (Life Technologies, Carlsbad, CA, United States). Samples were mounted in mounting medium containing 4,6-diamidino-2-phenylindole (DAPI) for nuclear staining, purchased from Nakalai tesque (Kyoto, Japan). Samples were observed with an Olympus fluorescence microscope IX83 (Olympus, Tokyo, Japan) or CellInsight CX5 (Thermo Fisher Scientific, Waltham, MA, United States). The following primary antibodies were used: mouse anti-Pax7 (sc-81648, used at 1:500 dilution) and rabbit anti-MyoD (sc-760, used at 1:1000 dilution) antibodies were purchased from Santa Cruz biotechnology (Santa Cruz, CA, United States). The mouse anti-MyHC antibody (MF20, MAB4470, used at 1:1000 dilution) was purchased from R&D Systems (Minneapolis, MN, United States). The rabbit anti-NG2 Chondroitin Sulfate Proteoglycan Antibody (AB5320, used at 1:250 dilution) was purchased from EMD Millipore (Burlington, MA, United States). The rat anti-CD140b (PDGFRβ) antibody (14-1402-82, used at 1:250 dilution) was purchased from Thermo Fisher Scientific (Waltham, MA, United States). The rat anti-CD140a (PDGFRα) antibody (135901, used at 1:250 dilution) was purchased from BioLegend (San Diego, CA, United States).

### Muscle Tissue

Skeletal muscle tissues used in the current study were human semitendinosus (ST) and mouse TA. Human ST were collected from patients undergoing reconstructive surgery following anterior cruciate ligament rupture. Mouse TA were collected from C57BL/6J mice.

### Inducing Differentiation

To investigate the myotube formation potential of isolated cells, muscle differentiation was induced on a matrigel-coated dish. Cells were expanded with GM and then cultured with differentiation medium (DM, 5% horse serum and 1% PS in DMEM).

### Statistical Analysis

For statistical comparisons of two conditions, Student’s unpaired, two-tailed, *t*-test was performed using GraphPad Prism (version 8). For all statistical tests, *p* < 0.05 was regarded as statistically significant. All error bars represent standard deviations.

### Ethics Statement

The Experimental Animal Care and Use Committee of Nagasaki University and Kumamoto University approved all animal experimentation (Ref. Nos. 1203190970 and A30-098). The use of human tissue samples was approved by the Nagasaki University Ethics Committee (Ref. No. 16052306).

## Results and Discussion

Efficient MuSC sorting is essential for the investigation of skeletal muscle regeneration and homeostasis in both, basic science and translational applications. This study presents a new method for MuSC isolation using basic laboratory equipment. This method reduces the procedure time to 2.5 days to purify an abundant number of myoblasts positive for Pax7 and/or MyoD (Figures 1C–E). Indeed, the major advantage of this method is “simplicity and timesaving.” Although the obtained cells are a limited population with low potential of attachment, it can be one of the options for isolating myogenic cells besides antibody-labeled cell sorting methods.

The pre-plate technique is based on the differences in adhesion characteristics of fibroblastic cells and MuSCs (Richler and Yaffe, 1970; Rando and Blau, 1994). At the first pre-plating step, most fibroblasts are trapped on the collagen-coated dish (Figure 1B). After 2 days of cell expansion in GM, fibroblasts can still be observed. To further purify MuSCs, we perform the additional re-plating step. At this point, most MuSCs are activated under the stimulation of GM and become much more adherent compared to quiescent MuSCs. In the re-plating step, the five-minute-incubation traps adherent cells mainly comprised of non-satellite cell populations including fibroblasts, pericytes/mesangioblasts, and mesenchymal progenitors, positive for NG2, PDGFRα and/or PDGFβ (Figure 1C; Uezumi et al., 2010; Judson et al., 2013).

This protocol can isolate purified MuSCs in 2.5 days. Unlike cell lines (e.g., C2C12 and L6), primary cultured MuSCs are more capable of differentiating, expressing differentiation markers, and fusing with each other during prolonged days of culture, even in GM. Here, we shortened the entire process by employing the re-plating step, enabling experimental applications to test MuSC proliferation with reduced influence of self-differentiation.

The pre-plate technique has been used for more than a decade (Gharaibeh et al., 2008), and has been improved in recent years (Xu et al., 2018). Many pre-plate techniques, including those with modifications, are already used in laboratories. As different from other existing pre-plating methods (Arsic et al., 2008; Gharaibeh et al., 2008; Li et al., 2010; Xu et al., 2018), our method does not need to complete the MuSC purification at the initial cell exposure to growth medium (GM), where cells are frequently lost. In our method, MuSCs can be expanded first, and then purified in the re-plating step. These procedures ensure the MuSC acquisition even from a small amount of muscle tissues. The re-plating step can be also inserted to activated and proliferative MuSCs. Accordingly, our method is used as “add-in” for any pre-plating methods. This approach is applicable to both pre-plating techniques and single-fiber techniques (Ono et al., 2012) where fibroblast contamination obstructs experiments after MuSCs migrate to the bottom of the dish.

**FIGURE 2:**
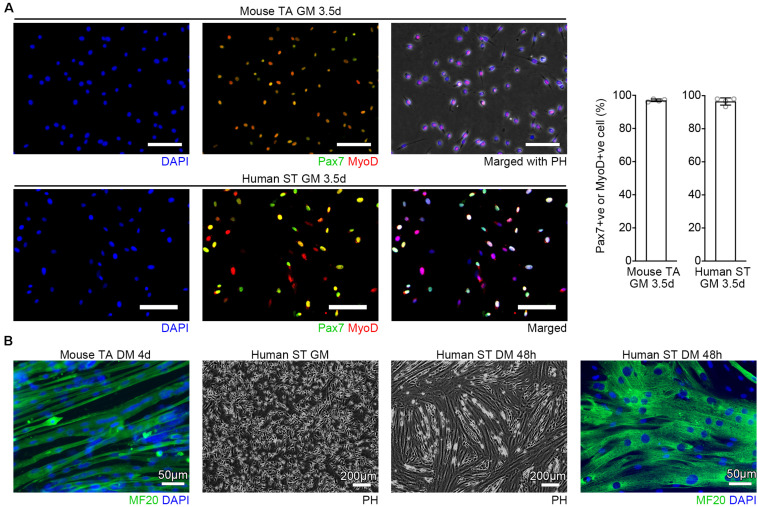
Verification of the purity. **(A)** Representative images of purified MuSCs cultured in GM and quantification of Pax7 or MyoD positive cells. Cells from mouse TA or human ST were immunostained with Pax7 (green), MyoD (red), and DAPI (blue). *n* = 4 each, Scale bars = 100 μm, Error bars = SD. **(B)** Representative images of purified MuSCs cultured in GM and DM. Cells from mouse TA or human ST muscles were immunostained for MF20 (green) combined with DAPI (blue) staining. After the human cells reached the confluence, the cells were induced to differentiation in DM for 48 h.

The protocol described here is applicable for various types of skeletal muscles of humans or mice. We confirmed that purified MuSCs were able to undergo myogenic differentiation as efficient as FACS-sorted cells (Kitajima and Ono, 2018) when exposed to DM (Figures 2A,B). Other than human ST and mouse TA tissues, we have also isolated MuSCs from the buccinator and masseter of human subjects, and the masseter, trapezius, supraspinatus, triceps, gastrocnemius, bulbospongiosus, and levator ani of C57BL/6J mice, using this method (data not shown). Because the protocol can isolate MuSCs from small amounts of muscle, it could be used, for example, to investigate differences between fast glycolytic muscles and slow oxidative muscles where the muscle volume obtained from a single mouse is limited.

Quiescent state of satellite cells is a key to maintain the stem cell function for population expansion *in vivo* when transplanted into recipient muscles (Collins et al., 2005). Although our modified pre-plating method is useful to obtain MuSCs from a limited amount of biopsy from the patients, it breaks the satellite cell quiescence during isolation. However, our method may be applicable for cell-therapy to treat muscle diseases by combining with emerging techniques controlling the cellular quiescence depth (Gilbert et al., 2010; Kwon et al., 2017; Fujimaki et al., 2019). In addition, our method may also be applied to the validation of muscle lineage cells derived from iPS cells.

FACS-sorting is a well-established method to isolate MuSCs. However, our method has advantages given its simplicity and the lack of cellular damage which result in higher yield. Our method can be one of the options for isolating myogenic cells besides antibody-labeled cell sorting methods.

Choosing the right experimental application would accelerate research, and the modified pre-plating method could be an alternative to FACS for isolating MuSCs in some experimental cases.

## Conclusion

This protocol enables MuSC isolation from human/mouse skeletal muscle tissue using basic laboratory equipment that is easily available. Furthermore, the rapid and high yield MuSC isolation achieved here allows us to perform further experimental applications to investigate muscle regeneration. We believe that this method provides the scientific community with an efficient approach to isolate MuSCs in terms of cell yield, simplicity, and cost-effectiveness.

## Data Availability Statement

The datasets generated for this study are available on request to the corresponding author.

## Ethics Statement

The studies involving human participants were reviewed and approved by Nagasaki University Hospital Clinical Research Ethics Committee. Written informed consent to participate in this study was provided by the participants’ legal guardian/next of kin. The animal study was reviewed and approved by the Experimental Animal Care and Use Committee of Kumamoto University.

## Author Contributions

KY conceived, designed, and performed the experiments, assembled the figures, analyzed the data, and wrote the manuscript. YK performed the experiments, analyzed the data, and wrote the manuscript. NO, KC, and AY provided analytical tools and gave technical support. YO designed the experiments, assembled the data, and wrote the manuscript. All authors discussed the results and commented on the manuscript.

## Conflict of Interest

The authors declare that the research was conducted in the absence of any commercial or financial relationships that could be construed as a potential conflict of interest.
